# The Effect of Increasing Topsoil Disturbance on Surface-Active Invertebrate Composition and Abundance under Grazing and Cropping Regimes on Vertisols in North-West New South Wales, Australia

**DOI:** 10.3390/insects11040237

**Published:** 2020-04-10

**Authors:** Johnson N. Nkem, Lisa Lobry de Bruyn, Kathleen King

**Affiliations:** 1 African Climate Policy Center, United Nations Economic Commission for Africa, Addis Ababa 1000, Ethiopia; nkem@un.org; 2 School of Environmental and Rural Sciences, University of New England, Armidale, NSW 2351, Australia; k.king8@bigpond.com

**Keywords:** agricultural intensification, invertebrate abundance, invertebrate composition, ants, soil health, Vertisols

## Abstract

Agricultural intensification practices involve varying degrees of disturbance to the soil ecosystem. This study evaluated six agricultural management regimes with increasing levels of topsoil disturbance, on the composition and abundance of surface-active invertebrates on Vertisols at a sub-catchment scale. Two grazing (native and introduced pastures), and four cropping (combining short and long fallow, with zero and conventional tillage) management regimes were examined. Surface-active invertebrates were collected seasonally with pitfall traps over 2 years (8 seasons), and identified to order, while ants (Formicidae) that comprised 47% of total invertebrates collected, were identified to genera. Season had a significant effect on ant abundance and number of genera recorded with higher abundance and twice the number of genera in summer than all other seasons. Ants, particularly *Iridomyrmex*, were mainly active in summer, while other invertebrates especially Coleoptera, were more active in winter. Surface-active invertebrates were 30% more abundant in grazing than cropping land use types. Native pasture, with little surface soil disturbance, recorded the highest number of invertebrates, mainly ants, compared to other agricultural management regimes. Coleoptera and Dermaptera were higher in abundance under conventional tillage compared with those agricultural management regimes that disturb the topsoil less. Optimizing surface-active invertebrate activity on Vertisols for most taxa will require reducing topsoil disturbance. However, the research findings also suggest that the impact of agricultural management regimes on invertebrate activity was difficult to predict with any certainty as the three main ant genera, and most abundant invertebrate collected, did not respond in a consistent manner.

## 1. Introduction

Soil health decline and invertebrate biodiversity loss are common symptoms of unsustainable soil management practices, especially under conventional agriculture with high inputs involving heavy use of agrochemicals, and frequent disturbance of the surface soil layer (top 0–20 cm) [1,2,3]. The lack of consideration of collateral impacts, due to agricultural intensification, on biota constitutes a serious risk to sustainability of the system and potential loss of ecosystem functioning [4,5,6]. For instance, the decline in European bat population [7], and native bees involved in crop pollination in the central valley coastal range of California [8] are good examples of the consequences of agricultural intensification on single vertebrate and invertebrate taxa, respectively, but significantly less is known about effects on community structure and seasonal activity patterns particularly of invertebrates under such systems.

A debate for some time has been on the role of invertebrate biodiversity per se over functional diversity in understanding the role of invertebrates in maintaining a healthy ecosystem in agricultural systems [9,10,11,12]. Hence the need to examine more closely the agricultural management practices and their impacts on invertebrate composition and activity in order to determine which taxa are able to persist in an agricultural system. While invertebrates may be regarded as pests or vectors of diseases to crops and animals [13,14], there is also compelling evidence of their role in the productivity and sustainability of the soil system [12,15,16,17,18,19,20], especially in ecosystem processes such as nutrient cycling, and soil bioturbation [21,22,23,24].

Scientists working in agricultural systems are seeking to address land degradation problems through modification and improvement of agricultural management practices [25,26,27,28,29,30,31,32], that combine sustainable farming and improved ecosystem functioning. Some of these adjustments to land management practices have contributed to increased activity and abundance of certain invertebrates, while others have resulted in further declines in invertebrate activity and simplification of community structure [27,28,29,33,34,35,36]. Determining the relationship between specific agricultural practices and associated level of soil disturbance on invertebrate activity and especially of taxa that interact with the soil to depth (greater than 20 cm) under agricultural practices pose important research questions. Furthermore, the combination of activities involved in an agricultural practice and the spatial and temporal nature of the soil disturbance associated with the practice, may affect invertebrate taxa differently. Thus, to identify degree of soil disturbance on invertebrate communities under particular agricultural management practices requires examination of the various components involved such as frequency and depth of tillage operations at time of seeding versus how long the soil is cropped or left undisturbed i.e., the length of fallow as they would occur in a commercial farm. Fallow in this context refers to land that has been left undisturbed in the current crop season to improve the productivity of the land by conserving soil moisture.

Vertisols are used for agriculture in many regions of the world including Australia, and covers 88 Mha or 11.8% of Australia’s land surface, with 17 Mha in New South Wales [37]. Despite the agronomic importance of Vertisols and the growing interest of land managers in invertebrates and their role in soil health and sustainability [38,39,40], there have been few extensive field studies examining quantitatively the levels and nature of surface-active invertebrate abundance on this soil type on commercial farms, particularly over a range of agricultural management regimes. Previous studies in Australia have indicated that Vertisols have recorded low levels of surface active soil invertebrates and fewer ant species that are likely to interact with the soil and perform functions in the soil [41] when compared to other soil types, such as Grey Sodosols and Yellow Kandosols in the central wheat-belt of Western Australia that recorded up to 30 ant species in farmed soils [33]. A study examining microbial biomass and free-living nematodes in cropped Vertisols came to the conclusion that biological activity in these soils was low, but had not examined surface active invertebrate taxa or their seasonal variations in abundance [42].

This study’s main aim was to evaluate the effect of topsoil disturbance on surface-active invertebrate’s relative abundance and composition through examination of six agricultural management regimes with increasing frequency and intensity of soil disturbance, all located on Vertisols [43]. The specific research questions were under increasing frequency and intensity of topsoil disturbance:

(1) Do invertebrate taxa and their levels of surface-active abundance vary seasonally (winter, spring, summer and autumn)?
(2) Do invertebrate taxa and their seasonal abundance vary under grazing and cropping land use types?
(3) Were there fewer invertebrate taxa and lower abundance where fallow length is shorter and tillage more intense?

## 2. Materials and Methods

**Figure 1:**
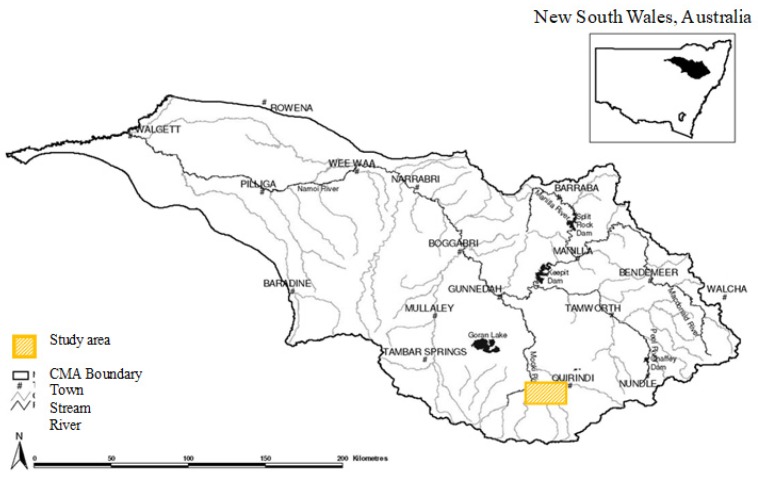
Location of study sites in Namoi catchment, New South Wales, Australia.

The study sites were located between Quirindi and Spring Ridge, New South Wales, Australia (longitude 150°15′–150°40′ E, latitude 31°20′–31°45′ S), an area predominantly involved in dryland cropping (18%) and livestock grazing (61%), and part of the Namoi Catchment [37] (Figure 1).

The rainfall distribution in the study region is bimodal with sufficient rainfall to plant winter and summer grain crops of wheat and sorghum, respectively. The mean annual rainfall recorded during the study period (over two years) was 880 mm with most of the rain falling over the summer period (December–February). The annual rainfall for 1999 was 787 mm, and 973 mm in 2000, which was 29% higher than the previous 10-year average of 755 mm per annum. Comparing the annual rainfall of 2000 to the long-term average data taken from the Australian Bureau of Meteorology shows that it was at the wetter end of the distribution (9th decile was 916 mm per annum, n = 120 years).

Six agricultural management regimes on Vertisols [43] were selected belonging to two major land use types (grazing and cropping). Vertisols are clay soils (>35% clay) with gradational texture profile and shrink-swell properties, which cause deep and wide cracking on drying, as well as self-mulching to depths of 5 cm. Lenticular structure and slickensides are diagnostic features of these soils. This soil type can vary in color—black, brown, grey and red and range from strongly acid to highly calcareous, but in this study area the soil type varied from grey to black and were predominantly neutral to mildly alkaline with a typical soil profile included in Table S1. The six agricultural management regimes selected represented an increasing topsoil disturbance based on their frequency of use (i.e., how often the land is cropped or grazed) and the intensity of tillage (number of tillage operations) (Table 1). In its natural state a Vertisol soil is under native grasses (often dominated by plains grass (*Austrostipa aristiglumis*), yadbila or coolabah grass (*Panicum queenslandicum*) or Queensland blue grass (*Dichanthium sericeum*)) with very little tree cover. Under this topsoil disturbance typology, it was characterized that short fallow conventional tillage (SFC) experienced the greatest frequency and intensity of soil surface disturbances through tillage, frequency of cropping and consequently shorter periods of fallow. Therefore SFC was classified as the most intensive agricultural management regime, while native pasture (NP), with its low stocking rates and long rest periods between grazing was the least intensive agricultural management regime in the typology and likely to experience the least topsoil disturbance (Table 1). The six agricultural management regimes thus examined are represented from least to most intense soil surface disturbance (0–20 cm depth) based on management practices (Table 1). These comprised of grazing land use under native (NP) or introduced (IP) pastures (hence not tilled, except when pasture established in IP and not fallowed); and cropping land use that was comprised of 2 agricultural management practices combining length of fallow (frequency of disturbance, short or long) and intensity of tillage (degree of disturbance, zero or conventional) combined in 4 ways, short fallow conventional tillage (SFC), long fallow conventional tillage (LFC), short fallow zero tillage (SFZ), long fallow zero tillage (LFZ) (Table 1).

Fallow refers to a piece of land that is normally used for farming but that is left with no crops on it for a period of time. Frequency of use was higher under short fallow where the separation between crops was between 6 to 9 months, while in long fallow, it was typically 12 to 18 months in one crop-fallow rotational cycle. Fallow practices are usually based on the need to conserve soil moisture for future crops, even though through extended fallow periods from cropping, other forms of land degradation may result such as increased groundwater recharge resulting in increased risk of soil salinity, and soil loss by water erosion as a consequence of low soil cover. Zero tillage is a cropping practice where there are no tillage operations before seeding, and weed control is through herbicide application and usually stubble from the previous crop is retained, whereas under conventional tillage, the soil is disturbed by ploughing to a depth of 15–20 cm prior to seeding at least several times. In summer, sorghum (December planting) was grown and in winter, wheat (May–June planting) was grown with either long or short fallow phases depending on number of crops grown. There is a complete rotational cycle under each agricultural management regime (Table S2) from least to most disturbance of the soil surface (native pasture (NP), introduced pasture (IP), long fallow zero tillage (LFZ), short fallow zero tillage (SFZ), long fallow conventional tillage (LFC), short fallow conventional tillage (SFC)). All the sites were located on commercial farms that had a minimum of five years continuous history of the specified agricultural management regime and undertaken by the same land managers or farm owners.

There were six agricultural management regimes of commercial farmers’ fields and they were all initially replicated four times. However, over the course of the study two fields selected for LFZ management regime no longer complied as they were cropped too frequently, and cultivated more frequently than was expected of long fallow zero tillage. These fields were reclassified to SFC. Thus, in total, there were 24 farmers’ fields sampled for surface-active invertebrates on 8 separate occasions, once in each season (winter (June–August), spring (September–November), summer (December–February) and autumn (March–May)), and repeated in another calendar year, hence giving a total of 192 sampling occasions. Each replicate of a field was independent of other replicates as sufficient distance (often tens of kilometers) separated replicates.

Mapping of soil invertebrate activity was considered as it reflects more closely resident taxa but it is very time consuming, and therefore not undertaken [33]. Pitfall traps, a widely-accepted technique for collecting surface-active invertebrates, especially ants, were made from plastic containers (50 mm in diameter and 75 mm deep) placed in a PVC plastic sleeve flush with the soil surface, and the technique allowed the assessment of commercial farm sites concurrently over a 72 h period (including day and night) [44,45,46,47,48,49]. The sampling of surface active invertebrates occurred within the center of each field to avoid edge effects in a 50 by 50 m sampling area divided into 5 by 5 (10 by 10 m) grids. In each of the 50 by 50 m sampling areas there were 20 pitfall traps. The pitfall traps were arranged as follows. A pitfall trap was randomly installed within a 0.5 m radius of each corner of a 10 by 10 m grid over the 50 by 50 m sampling area, with the same 5 outer quadrats excluded from sampling. Hence, 160 pitfall traps were collected from the same sampling area in the farmer’s field, by sampling once each season and repeating in a separate calendar year. A total number of 3840 pitfall traps were collected over the sampling period (24 fields × 20 pitfall traps/field × 8 sampling occasions/field = 3840). To facilitate sampling and minimize frequent digging and creating any disturbance to the trap area, permanent PVC sleeves (50 mm diameter × 100 mm long) were placed into the soil several days before the commencement of sampling. During each sampling period, debris was removed from the PVC sleeves, and a plastic container was inserted without causing additional disturbance. The pitfall traps were placed flush with the soil surface, 3/4 filled with 70% ethyl alcohol and 10% glycerol mixture and left open for three consecutive days and nights, after which the pitfall traps were collected, and their contents sorted and identified. All 24 sites were sampled concurrently.

While most invertebrates were identified to Order [50], Coleoptera were also identified to Family level, and ants were identified to Genera because the taxonomic expertise was available, and also because their expected numerical abundance in pitfall traps made it possible to evaluate seasonal changes and invertebrate community responses to agricultural management regimes at this taxonomic level. Another consideration was identification to species level that would have given finer granularity to the impacts of agriculture at that level. However, the objectives of the wider study, which this work formed part of were looking to examine fluctuations at a broader taxonomic level that farmers could use, given they have limited taxonomic expertise. In addition, as this work formed part of a larger study, it was logistically impossible for a single person to sort and identify all specimens to species level over the project duration.

The collection of invertebrates in pitfall traps will be referred to as abundance data, and the data analyzed were the mean value of the replicated land use types or agricultural management regimes based on total count of invertebrates and invertebrate taxa collected from 20 pitfalls per field per sampling period (of which there were 8 sampling occasions per field). The invertebrate data were transformed by Log (n + 1) as this normalized the data. Then Analysis of Variance (ANOVA) in StatView [51] was used to examine the following relationships between total abundance of surface-active invertebrates, and of invertebrate taxa with:

(1) Seasonality (winter, spring, summer and autumn) within year of collection,
(2) Land use type (either cropped or grazed) and
(3) Agricultural management regimes (NP, IP, LFZ, SFZ, LFC, SFC) across a topsoil disturbance typology (Table 1).

Seasonality within year of collection as factors were blocked for the land use type and agricultural management regimes in a two-way repeated measures ANOVA. Statistical differences were significant at *p* < 0.05 unless otherwise stated. The number of replicates upon which the mean was based, and the standard error of the mean were stated in the appropriate tables and figures. In addition, ranking of number of ant genera in agricultural management regimes was conducted in Excel, and blocked by season to avoid confounding effects.

## 3. Results

The results focus on the three research questions in sequence and examine them in order of total invertebrate abundance, followed by those invertebrate taxa that recorded greater than 1% of the pitfall trap collection, and with soil interactions (e.g., life cycle stage, habitation in soil or functional role) and finally the most dominant invertebrate order collected—Ants. Complete invertebrate data collected are presented in Tables S3 and S4.

Total abundance of invertebrate taxa recorded over the eight sampling periods in two calendar years was dominated by a few taxa, with ants constituting the dominant invertebrate from all the sites, followed by Diptera and Coleoptera (Table 2).

Other surface-active invertebrate taxa that were trapped in lesser numbers were Araneae Hemiptera and Acarina (Table 2). Meanwhile, 9 taxa recorded less than 1% of the pitfall catch over the eight sampling periods (Table 2).

**Figure 2:**
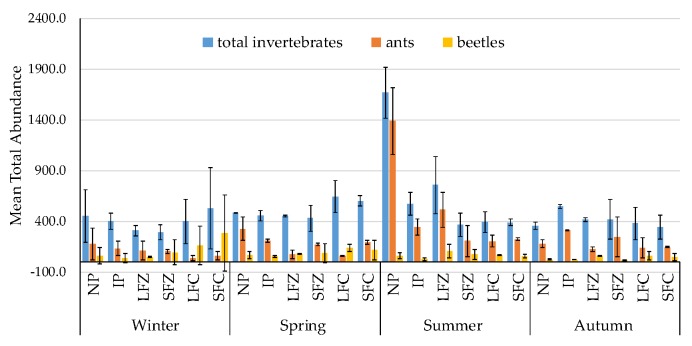
Seasonal variations in mean total abundance of invertebrates collected (total invertebrates, ants and beetles per field) during the study period under agricultural management regimes. Each data point was the total abundance of 20 pitfall traps per site, with each field (n = 24) sampled eight times. The mean for a specific treatment was based on the number of replicates for that particular grouping. Total number of sampling occasions = 192. NP = native pasture, IP = introduced pasture, LFZ = long fallow zero tillage, SFZ = short fallow zero tillage, LFC = long fallow conventional tillage, SFC = short fallow conventional tillage.

Total invertebrate abundance varied under the agricultural management regimes with native pasture (NP) consistently recording the highest number of invertebrates in summer, while long fallow zero tillage (LFZ) had the second highest number of invertebrates recorded, and introduced pasture (IP) had the third highest number of invertebrates recorded in the same season (Figure 2). Meanwhile, the peaks in numbers of invertebrates collected over winter were mainly recorded in short fallow conventional tillage (SFC), and were the result of a high proportion of *Staphylinidae* beetles collected, with troughs in beetle abundance recorded in autumn (Figure 2). Overall, the remaining agricultural management regimes had similar total invertebrate abundance, despite the seasonal variability (Figure 2).

**Figure 3:**
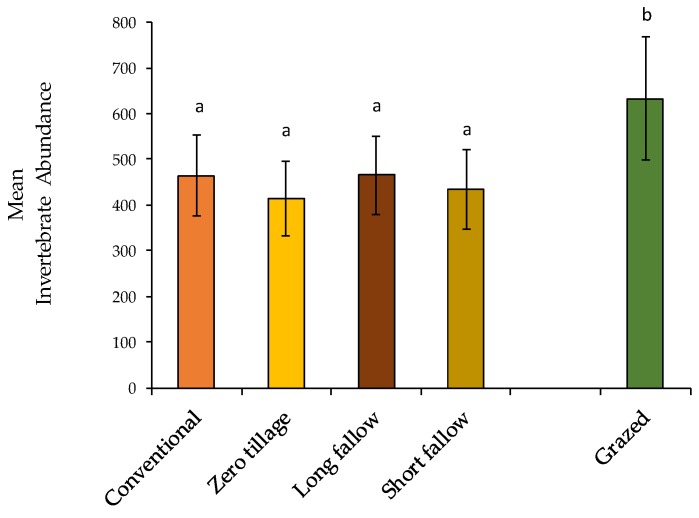
Variations in mean total invertebrate abundance (mean total per field) under tillage, fallow regimes under cropping land use type and grazing land use type over study period. Each data point was the total abundance of 20 pitfall traps per field, with each field (n = 24) sampled eight times (once per season) over two years. The mean for a specific treatment was based on the number of replicates for that particular grouping i.e., grazing (n = 64) or cropping (n = 128). Total number of sampling occasions = 192. Bars signify ± SEM.

Total numbers of invertebrates collected were consistently higher under grazing compared with cropping land use types (F_1,1_ = 13.042, *p* = 0.0004). There were similar numbers of invertebrates collected under zero and conventional tillage regimes as well as under short and long fallow (Figure 3).

Tillage effects on numbers of surface-active invertebrates collected corresponded to intensity (tillage), and frequency (length of fallow) of topsoil disturbance when compared with grazing land use type that is not tilled or fallowed (Figure 3). The grazing land use type (introduced and native pasture combined), although displaying greater variability in invertebrate numbers, recorded on average 30.5% more invertebrates than both conventional (F_1,2_ = 6.487, *p* = 0.0012) and zero tillage practices (F_1,2_ = 6.488, *p* = 0.0045) under cropping land use types. Similarly, grazing land use types (which experiences no fallow periods) recorded significantly higher invertebrate abundance, by 29%, compared with long (F_1,2_ = 6.488, *p* = 0.0047) and short fallow (F_1,2_ = 6.487, *p* = 0.0011) under cropping land use type (Figure 3).

### 3.1. Non-Formicidae (Other Invertebrates excluding Ants) Abundance

**Figure 4:**
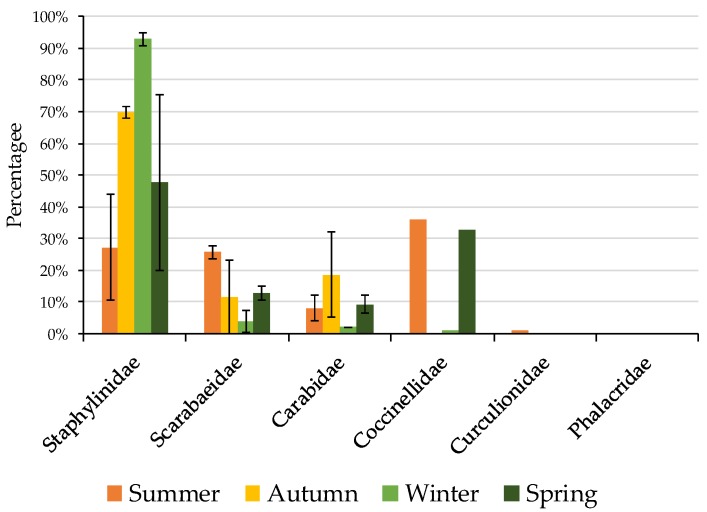
Proportion of Coleoptera Families (mean total per field) recorded seasonally and averaged over all land use types, and agricultural management regimes during the study period. Each data point was the total abundance of 20 pitfall traps per field, with each field (n = 24) sampled on eight occasions, once in each season and repeated in the following calendar year.

Non-Formicidae invertebrates were more abundant in winter and spring than autumn and summer (F_1,3_ = 3.254, *p* = 0.0229). In particular, the numbers of beetles (Coleoptera) collected were higher in winter compared with autumn (Table 3). Examination of Coleopteran Families showed a dominance of *Staphylinidae* in winter and autumn, and in spring and summer a greater proportion of *Scarabaeidae*, with a small proportion of *Carabidae* caught (Figure 4). In conventional tillage (mean = 69%, 43%–90% range) a higher proportion of non-ant invertebrate specimens were recorded compared with grazing (mean = 46%, 17%–67% range), and zero tillage (mean = 58%, 32%–70% range). Fallow practices, under conventional cultivation (C) whether short (SF) or long (LF) had no significant effect on non-ant abundance with both LFC and SFC recording higher non-ant abundance than short fallow and zero tillage (SFZ), but not LFZ (F_1,5_ = 1.941, *p* = 0.0073).

The non-Formicidae orders responded inconsistently to combinations of land use types and agricultural management regimes. Cropping recorded higher numbers of beetles than grazing land use (F_1,1_ = 4.223, *p* = 0.0051) (Table 4), with conventional tillage also recording significantly higher beetle numbers than grazing land use type (F_1,1_ = 4.223, *p* = 0.0051). Short fallow and long fallow under conventional tillage recorded similar levels of beetle abundance as did the two types of fallow under zero tillage (Table 4). Finally, SFC regime was significantly higher in beetle numbers than native pasture (NP) (F_1,5_ = 1.719, *p* = 0.0133) and introduced pasture (IP) (F_1,5_ = 1.719, *p* = 0.0440) (Table 4). Araneae, mostly wolf spiders (*Lycosidae*), were lowest in abundance during winter while greater numbers were recorded in spring and summer (F_1,3_ = 9.626, *p* = 0.0001) (Table 3). Examining the combinations of agricultural management regimes, it was found that introduced pasture had recorded significantly more spiders than the other agricultural management regimes (F_1,5_ = 3.245, *p* = 0.0080) (Table 4). Acarina (mites), like Araneae, experienced higher levels of abundance under introduced pasture (F_1,5_ = 2.049, *p* = 0.05), but were more abundant in winter (Table 3).

### 3.2. Formicidae Composition and Abundance (Ants)

**Figure 5:**
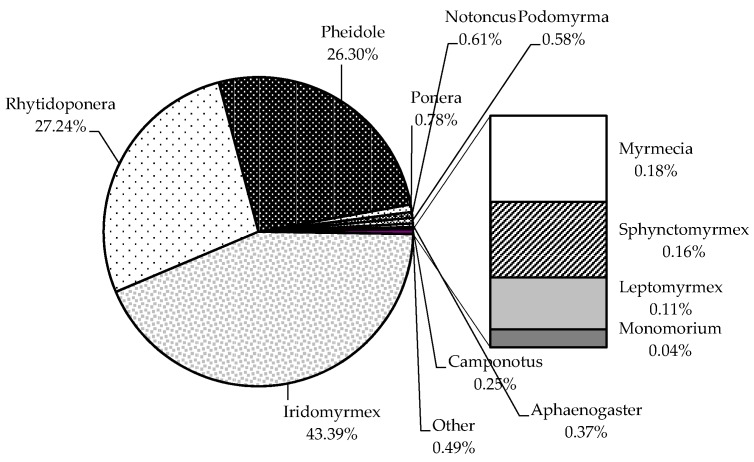
Proportion of mean ant abundance (mean total per field) recorded at all seasons, land use types and agricultural management regimes during the study period. Each data point was the total abundance of 20 pitfall traps per field, with each field (n = 24) sampled on eight occasions, once in each season and repeated in the following calendar year. The mean for a specific treatment was based on the number of replicates for that particular grouping i.e., grazing (n = 64) or cropping (n = 128). Total number of sampling occasions = 192.

Season had a significant effect on ant abundance and composition with higher numbers of ants collected in summer than all other seasons (F_1,3_ = 15.395, *p* = 0.001) (Table 3). In summer, twice the number of ant genera were recorded compared with winter (Table 3). Three ant genera (*Iridomyrmex*, *Rhytidoponera*, and *Pheidole*) were caught in all pitfall traps, and these genera comprised 43, 27 and 26% of the total pitfall catch, respectively (Figure 5). The remaining 4% of the pitfall catch was represented by 9 genera (Figure 5).

**Figure 6:**
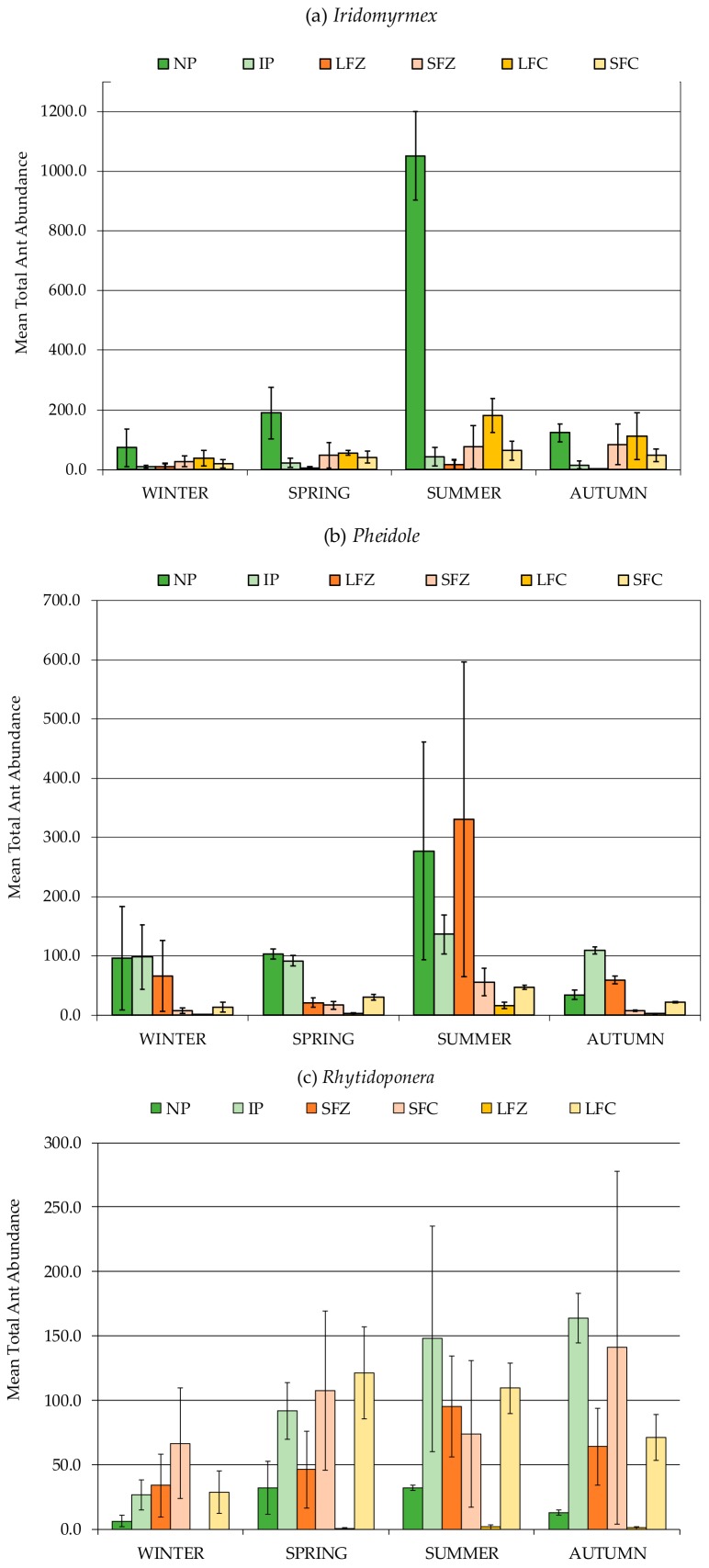
Mean abundance (mean total per field) of three dominant ant genera from most to least abundant: (**a**) *Iridomyrmex,* (**b**) *Pheidole,* (**c**) *Rhytidoponera,* and under six agricultural management regimes during the study period. Each data point was the total abundance of 20 pitfall traps per field, with each field (n = 24) sampled on eight occasions, once in each season and repeated in the following calendar year. The mean for a specific agricultural management regime was based on the number of replicates for that particular grouping i.e., a specific agricultural management regime such as native pasture monitored in winter (n = 8). Total number of sampling occasions = 192. Bars signify ± SEM. NP = native pasture, IP = introduced pasture, LFZ = long fallow zero tillage, SFZ = short fallow zero tillage, LFC = long fallow conventional tillage, SFC = short fallow conventional tillage.

Ant numbers were also much higher under grazing than cropping land use types (F_1,1_ = 21.087, *p* = 0.0001) (Figure 2, Table 4). Higher ant numbers, especially *Iridomyrmex* (F_1,5_ = 15.312, *p* = 0.0001) and *Pheidole* (F_1,5_ = 5.767, *p* = 0.0040), were recorded under native pasture (NP) (Figure 6a,b) compared with the other agricultural management regimes (Table 4). *Pheidole* were more abundant in the grazing land use type compared with cropping (Table 4). Nevertheless, high numbers of *Pheidole* were recorded in LFZ, in summer, comparable to those numbers recorded under grazing land use type (Table 4, Figure 6b). In contrast to *Iridomyrmex* and *Pheidole*, *Rhytidoponera* showed no seasonal preference and were equally abundant over summer, autumn and spring with variable numbers recorded over winter (Table 3, Figure 6c). There were no clear statistical differences in activity of *Rhytidoponera* between cropping and grazing land use types (Table 4). However, low numbers of *Rhytidoponera* were recorded for native pasture (NP) and long fallow conventional tillage (LFC) compared with other cropping and grazing systems (IP, SFZ, SFC, LFZ) (Table 4, Figure 6c).

## 4. Discussion

Combining conservation and production goals, especially on prime agricultural land like Vertisols, remains a significant challenge. The native pasture grazing systems were higher in surface-active invertebrate abundance than cropping land, confirming similar observations of native pasture on other soil types [34,52,53,54]. Introduced pastures recorded levels of invertebrate abundance similar to that of cropping-based management practices, with some invertebrate groups such as Acarina recording higher levels of abundance under introduced pasture compared with all other agricultural management regimes (Table 3). It has been hypothesized that invertebrate numbers, especially in the soil surface, were affected by stocking rate of grazing animals [55,56,57,58]. Those areas with lower stocking rates, as in native pasture, would encourage greater invertebrate proliferation than under introduced pasture, which usually supports higher stocking rates than native pasture. Hence, the effects of higher stocking rates on surface-active invertebrate composition and abundance could be comparable to disturbance regimes under cropping systems.

By quantifying the levels of surface-active invertebrate activity on the basis of numbers collected, the greater presence of more numerically abundant taxa, such as ants, tends to obscure the effects of agricultural management regimes on less abundant but commonly recorded taxa such as Dermaptera or Araneae. Nevertheless, this situation was tempered by assessing invertebrate taxa separately, and therefore emphasizing the sensitivity of other invertebrate groups to seasonal changes and the associated disturbances within the agricultural management regimes.

Increased ant activities over summer have been widely reported in various parts of Australia [33,59,60] on other soil types, but there are few examples of such monitoring activities on Vertisols. In cropping systems, the absence of cultivation practices during summer periods especially under long fallow, could provide some opportunity for higher ant abundance, due to less likelihood of nest disturbance. According to the ant functional group nomenclature by Anderson [59] the ant genera, *Iridomyrmex*, are a dominant functional group. Therefore, where *Iridomyrmex* sp. were present in high numbers they can affect other ant species using their aggressive characteristics to easily out compete other ant species, thereby reducing their abundance [61,62,63]. Here, for example, *Iridomyrmex* may have reduced the prevalence of other ant groups, which were commonly recorded in those habitats where *Iridomyrmex* was absent. Body size is another factor that will influence abundance, with smaller-bodied ants as recorded in *Iridomyrmex* being more abundant than solitary foraging larger-bodied ants such as *Rhytidoponera. Pheidole*, a generalist functional group [59], that are highly adaptable, were abundant in the native pasture, while *Rhytidoponera*, an opportunist functional group [59], occurred mostly in the introduced pasture, and the more frequently cropped short fallow systems. In this study, a seasonal contrast in invertebrate abundance was observed, which was further amplified under certain agricultural management regimes. In particular, native pasture had the greatest contrast in invertebrate activity between warm and cool seasons mainly due to a peak in numbers of ants, especially *Iridomyrmex*, during summer.

Amongst the two broadest categories of invertebrates recorded during the study, ants peaked in abundance over summer while generally non-ant invertebrate groups peaked in abundance over winter. In addition, when ants were less active in winter compared with non-ant invertebrate groups, the non-ant invertebrate groups were more active. Hence the response of surface-active invertebrates to seasonal changes were complementary. The major groups of non-Formicidae with significant abundance during the study period were Coleoptera, Araneae and Acarina. *Staphylinidae* beetles that were recorded in high numbers, especially in SFC, would most likely be small detritovores, fungivores and small scale predators. Unlike ants, these arthropod groups occurred mostly in winter and spring with the least numbers in summer, except for Araneae. This result is particularly important in that invertebrate activities such as soil mixing, seed collecting or predation are not completely depressed or absent over the winter period. Although, there might not be complete complementarities in functions between ants and other invertebrate groups, the decline in activity in one group is compensated by the increased level of activity in another group thereby maintaining biological activities over the course of a year to varying degrees. The alternating peaks in activity between ants and non-ants, and their higher abundance under certain agricultural management regimes, could provide guidance as to when to sample for those particular taxa in agricultural systems.

Those invertebrates best able to survive, increasing topsoil disturbance, largely through cultivation or increased frequency of cultivation due to shorter fallow periods, therefore demonstrating greater resilience to perturbations would probably have greater mobility, and re-colonization potential or ability to avoid such disturbances with brood chambers (e.g., ants) well below the plough layer. Adult-winged invertebrates like beetles and flies, unlike most invertebrates dwelling in the upper 15 cm of soil, could avoid mechanical damage, and loss of nest openings or structures, although the soil-dwelling juvenile forms, such as Coleopteran larvae, would be affected.

Ants were more abundant in native pasture, while non-ant invertebrate groups, such as Coleoptera, were more commonly recorded in long fallow systems, either conventional or zero tillage. The contrasts in agricultural management regime preferences between ants and non-ants in cropping and grazing land use types is difficult to explain without a deeper understanding of the ecology of the taxonomic groups and also identification of taxa to a finer level of resolution. Habitat disturbance could be a factor for ants, as the construction of ant nests, whether large or small, will be disturbed in cropping systems involving conventional tillage, and even under high grazing frequency as in introduced pasture through cultivation when establishing a new pasture. A similar reduction in ant abundance along gradients of increasing agricultural disturbance was observed in a wet Costa Rican forest [64]. Other studies have demonstrated a similar influence of agricultural practices on invertebrate composition and activity in Australian soils [53,65,66]. Significant decreases in the abundance and diversity of invertebrate communities have been reported under high input cropping systems [34]. However, in our study the patterns of abundance under agricultural management regimes were inconsistent, with some invertebrates, like ants, specifically *Iridomyrmex* demonstrating greater abundance under native pasture, while other ants, such as *Rhytidoponera* did not (Table 4, Figure 6a,c). The greater abundance of *Iridomyrmex* under native pasture compared with other agricultural management regimes can be explained by the simplification of cropping habitats [29,67,68], and in introduced pasture a reduction in ground cover under frequent grazing [67]. However, in contrast, *Rhytidoponera* abundance was lower in native pasture and SFC, and greater numbers were recorded in IP, LFZ, SFZ and LFC agricultural management regimes. This particular result was not consistent with work examining the impacts of ground cover management on insect abundance that found in untilled areas where spontaneous vegetation could grow significantly more Formicidae and Coleoptera were recorded compared with the tilled treatments [68].

Cultivation practices often result in the reduction of invertebrate abundance [69,70,71,72] with reduced tillage having beneficial impacts on invertebrate populations [65,73,74,75,76,77,78]. In our research sensitivity of particular invertebrate taxa to topsoil disturbance, as expressed by a decline in abundance, as agricultural management regimes intensified such as greater frequency of cultivation or more frequent use (shorter periods of fallow), showed few consistent trends across invertebrate orders. While Coleoptera numbers were greater under conventional tillage, *Rhytidoponera* numbers were higher under short fallow, regardless of tillage intensity (Table 4). In a study on soil dwelling, Coleoptera community composition in maize the influence of tillage, fertilizer and weeding intensity were examined and there was a general decline in Coleoptera species diversity under conventional tillage, and under high weeding intensity, but no effect of fertilizer application [69] that was not demonstrated in this study. Possibly the larval stages of *Scarabaeidae* beetles would be susceptible to conventional tillage in autumn, before they emerge as adults in late winter to early spring.

The assemblages and abundance of particular invertebrate groups recorded under each agricultural management regime represents the availability of a niche in the agricultural system that allows them to thrive or not under a particular agricultural management regime. The stability of invertebrate community composition relates to the availability of a diverse number of niches in the soil that can sustain such communities and is viewed by some as a potential tool for monitoring changes in soil properties or the impacts of human activities [67,79,80,81]. Hence, to assess the impact of agricultural management regimes they need to be monitored reliably and efficiently. In addition, for any soil impacts to be mitigated or avoided will depend on gathering further understanding of how the soil ecosystem functions, especially quantifying the role of soil invertebrates in soil ecosystem processes such as bioturbation, water movement and soil structure formation [23,82,83,84,85].

## 5. Conclusions

Sustainable use of natural resources, specifically soil, and the increase of invertebrate activity by a range of taxa requires understanding the relationship between invertebrate taxa and topsoil disturbance in combination with agricultural management regimes before it is possible to trace changes in land management and detect human-induced disturbances of ecosystem processes. Improving invertebrate activity and the number of taxa under agricultural production regimes has beneficial consequences for economic productivity and the long-term sustainability of the soil ecosystem. Evaluating the changing pattern of invertebrate composition and activities as agricultural management regimes increase or decrease in their level of topsoil disturbance may signal a probable decline or improvement in soil condition. However, the practice of monitoring invertebrate abundance would be only possible with a mutual understanding between scientists and practitioners about the effect of agricultural management practices on taxa and if it is in a predictable way in an agricultural system. The research findings also suggest that the impact of agricultural management regimes on invertebrate activity was difficult to predict with any certainty as the three main ant genera, and most abundant invertebrate collected, did not respond in a consistent manner. While for non-ant fauna, greater activity was recorded under tillage with length of fallow being inconclusive as to its influence. Seasonal differences in invertebrate composition and abundance recorded in this study suggest that there are certain invertebrate taxa whose activities occur, at different times of the year, and could contribute a functional role either as predators, herbivores, detritivores or in mediating soil processes over a whole year if taxa are combined. In addition, to strengthen the potential for such monitoring a layperson’s guide to invertebrate collection and identification, along with ecological information on the functional role of taxa in the agroecosystem would be required.

## Figures and Tables

**Table 1:** Hypothesized overall topsoil disturbance typology in relation to agricultural management practices with native pasture (NP), introduced pasture (IP), long fallow zero tillage (LFZ), short fallow zero tillage (SFZ), long fallow conventional tillage (LFC), short fallow conventional tillage (SFC) based on typical regional practices.

Land Use Types	Agricultural Management Regime	Tillage Intensity (Number of Passes)	Length of Fallow (Months)	Stocking Density (dse/ha/yr)	Pesticide/Herbicide Spraying (Number of Passes)	Overall Level of Topsoil Disturbance
Grazing	NP	None	None	3	None	Low
	IP	None	None	9	None	Low
Cropping	LFZ	0	12–18	None	0–1	Low–Moderate
	SFZ	0	6–9	None	1–3	Moderate
	LFC	3	12–18	None	0–1	Moderate–High
	SFC	6	6–9	None	1–2	High

**Table 2:** Total number and relative abundance of invertebrate taxa, collected in pitfall traps over eight sampling periods in two calendar years (n = 3840 pitfall traps).

Taxa	No	%	Taxa	No	%
Hymenoptera (Ants only)	44,658	47.2	Diplopoda	855	0.9
Diptera	15,402	16.3	Dermaptera	812	0.9
Coleoptera	15,231	16.1	Blattodea	543	0.6
Orthoptera	4750	5.0	Thysanoptera	411	0.4
Hemiptera	3472	3.7	Chilopoda	166	0.2
Araneae	3467	3.7	Lepidoptera	129	0.1
Acarina	2960	3.1	Isopoda	117	0.1
Hymenoptera (no Ants)	1552	1.6	Mecoptera	34	0.04
Grand total	94,557				

**Table 3:** Mean abundance (standard error of mean) of invertebrate taxa recorded in pitfall traps (total number per field), across season and presented from most to least abundant. Each data point was the total abundance of 20 pitfall traps per field. Each field was sampled once in each of the four seasons and repeated in the following calendar year, n = 48. Within rows and category, means followed by different letters were significantly different at *p* < 0.05. Comparison of mean (Tukey’s HSD) were completed within the category of season. In addition, within rows, means followed by different letters were significantly different at *p* < 0.05.

Taxa	Season			
	Autumn	Winter	Spring	Summer
Formicidae (Ants)	192.9 b	100.3 b	180.5 b	455.8 a
	(23.2)	(21.3)	(24.3)	(93.8)
*Iridomyrmex*	68.2 b	30.6 b	61.3 b	248 a
	(14.8)	(9)	(12.7)	(70.9)
*Pheidole*	59.3 b	42.8 b	45.2 b	120 a
	(8.6)	(16.8)	(8.1)	(33.8)
*Rhytidoponera*	76.2 a	26.6 b	72.9 a	77.8 a
	(19.4)	(7.6)	(19.2)	(19.9)
Coleoptera	39.2 b	134.8 a	93.6 ab	61.1 ab
	(5)	(42.6)	(13.3)	(9.9)
Hemiptera	25.1	8.3	30	9
	(10.7)	(2.1)	(20.5)	(2.4)
Araneae	21.8 a	13.3 b	27.7 a	29.4 a
	(4.2)	(4)	(4)	(4)
Hymenoptera (excluding ants)	9.1 ab	3.0 c	13.6 a	6.7 bc
	(0.9)	(0.7)	(2.8)	(1)
Acarina	4.7 b	24.4 a	5.5 b	10.9 ab
	(0.9)	(9.7)	(1.2)	(2.3)

**Table 4:** Mean abundance (standard error of mean) of invertebrate taxa recorded in pitfall traps (total number per field), across land use and agricultural management regimes. Each data point was the total abundance of 20 pitfall traps per field. Each field was sampled eight times (once each season over two years), the number of replicates for each land use type or management regime is dependent on groupings. For each taxa, where there were significant differences (*p* < 0.05). Within rows and category, means followed by different letters were significantly different at *p* < 0.05. Comparison of mean (Tukey’s HSD) were completed within the category of land use type, and agricultural management regimes. NP = native pasture, IP = introduced pasture, LFZ = long fallow zero tillage, SFZ = short fallow zero tillage, LFC = long fallow conventional tillage, SFC = short fallow conventional tillage.

Taxa	Land Use Type				Management Regimes			
	Grazing	Cropping	NP	IP	LFZ	SFZ	LFC	SFC
Formicidae (Ants)	383.2 a	157.0 b	517.8 a	248.6 ab	206.6 ab	181.9 b	109.7 b	155.3 b
	(70.6)	(16.7)	(133)	(36.3)	(72.3)	(35)	(36.3)	(24.4)
*Iridomyrmex*	191.0 a	57.5 b	359.3 a	22.8 b	24.5 b	59.2 b	96.5 b	41.3 b
	(54.4)	(8.1)	(101)	(5.6)	(15.7)	(17.4)	(22.1)	(8.1)
*Pheidole*	118.6 a	32.5 b	128 a	109 ab	120 ab	22.2 bc	5.4 c	28.2 bc
	(22.3)	(9.4)	(38.8)	(22.5)	(71.4)	(4.9)	(2.9)	(3.8)
*Rhytidoponera*	64.1 a	63.0 a	20.8 bc	107.5 a	59.8 ab	97.3 ab	0.84 c	82.6 ab
	(13.5)	(11.3)	(5.3)	(24.3)	(16.5)	(28.7)	(0.3)	(21)
Coleoptera	45.1 b	100.7 a	53.7 b	36.5 b	74.5 ab	67.7 ab	106.7 a	127.5 a
	(5.5)	(17.1)	(9.8)	(4.8)	(13.7)	(18.3)	(18.5)	(42)
Araneae	29.0 a	20.0 b	23.8 ab	34.2 a	20.5 b	16.4 b	23.7 ab	19.9 b
	(3.1)	(1.7)	(3.4)	(5)	(4.4)	(1.9)	(4.4)	(2.8)
Acarina	18.2 a	7.9 b	8.7 b	27.8 a	2.3 b	5.3 b	8.3 b	11.4 b
	(4.3)	(3.2)	(1.7)	(8.1)	(0.9)	(1.8)	(2.8)	(8.2)
Hymenoptera (excluding ants)	9.5 a	7.4 a	9.7 ab	9.3 ab	10.4 ab	3.8 b	13.3 a	5.0 b
	(1)	(1.2)	(1.4)	(1.4)	(4.1)	(0.8)	(3.8)	(0.7)

## Supplementary Materials

The following are available online at https://www.mdpi.com/2075-4450/11/4/237/s1, Table S1: Typical Vertisol soil profile description, Table S2: Site level treatment according to season, Table S3: Mean abundance (standard error of mean) of invertebrate taxa recorded in pitfall traps (total number per field), across land use and agricultural management regimes. Each data point was the total abundance of 20 pitfall traps per field. Each field was sampled eight times (once each season over two years), the number of replicates for each land use type or management regime is dependent on groupings). For each taxa, where there were significant differences (*P* < 0.05). Within rows and category, means followed by different letters were significantly different at *P* < 0.05. Comparison of mean (Tukey’s HSD) were completed within the category of land use type, and agricultural management regimes. NP = native pasture, IP = introduced pasture, LFZ = long fallow zero tillage, SFZ = short fallow zero tillage, LFC = long fallow conventional tillage, SFC = short fallow conventional tillage, Table S4: Mean abundance (standard error of mean) of invertebrate taxa recorded in pitfall traps (total number per field), across season. Each data point was the total abundance of 20 pitfall traps per field. Each field was sampled once in each of the four seasons and repeated in the following calendar year, n = 48). Within rows and category, means followed by different letters were significantly different at *P* < 0.05. Comparison of mean (Tukey’s HSD) were completed within the category of season. Also, within rows, means followed by different letters were significantly different at *P* < 0.05.
