# Supplementary material for: The Effect of Increasing Topsoil Disturbance on Surface-Active Invertebrate Composition and Abundance under Grazing and Cropping Regimes on Vertisols in North-West New South Wales, Australia
Source: Insects. 2020 Apr 10;11(4):237. doi: 10.3390/insects11040237 (PMC7240756; doi:10.3390/insects11040237)
Supplement: Supplementary file 1 [file insects-11-00237-s001.pdf]

Table S1: Typical Vertisol soil profile description

SITE DETAILS

Site Location:	BVILLE - BLACK CK NR BVILLE RD
Profile Details:	Soil Landscapes of the Blackville 1:100 000 Sheet Survey (1000640), Profile 63, collected from a gully by Mr Robert Banks on 09 May, 1995
Map Reference:	MGA Grid Reference: Zone 56, 238755E, 6494889N. 8934 BLACKVILLE (1:100000) map sheet.
Physiography:	gully under littoral complex on alluvium, gravel lithology and used for volun./native pasture. Slope 1.0% (measured), local relief extremely low (< 9m), elevation 380.0 m. Surface condition is cracked, profile is imperfectly drained, erosion hazard is high, and no salting evident
Vegetation/Land Use:	limited clearing at the site, used for volun./native pasture, with cropping, volun./native pasture in the general area
Surface Condition:	cracked when described, expected to be cracked when dry, ground cover is 100%
Erosion/Land Degradation:	high; sheet erosion at site is moderate, partly stabilised; scald erosion at site is partly stabilised; tunnel erosion at site is evident, active; streambank erosion at site is evident, active; no salting evident
Soil Hydrology:	profile is moderately permeable and imperfectly drained, no free water, run on is high and runoff is high
Soil Type:	Epicalcareous Self-mulching Black Vertosol (ASC), Black Earth (GSG)
Base of observation:	soil continues
Profile Field Notes:	Site = Blackville TSR.

SOIL DESCRIPTION

**Layer 0**		
0.00 - 0.00 m		
	Coarse Fragments:	not evident,
**Layer 1**	Horizon: A1	
0.00 - 0.05 m	Texture:	heavy clay
	Colour:	very dark grey (brownish black) (5YR 3/1) [moist] with 0% unspecified mottles
	Structure:	strong pedality (polyhedral, 2 - 5 mm, fabric is smooth-faced peds), ped coatings are none
	Coarse Fragments:	not evident,
	Segregations:	not evident,
	Roots:	many (25-100/10x10cm) (Root size <1 mm),

	Soil fauna:	Activity is nil
	Cracks/Macropores:	Cracks are evident(width 10-20 mm), macropores are nil
	Moisture/Consistence:	dry,
	Erodibility Tests:	Crumb (EAT) test showed aggregates slake,
	Field chemical tests:	Field pH is 7.5 (Raupach), HCl test showed no effervescence, H2O2 showed no effervescence
	Sample taken:	disturbed
**Layer 2**	Horizon: B21	
0.05 - 1.40 m	Texture:	heavy clay
	Colour:	very dark grey (brownish black) (5YR 3/1) [moist] with 0% unspecified mottles
	Structure:	strong pedality (prismatic, 50 - 100 mm, fabric is smooth-faced peds), ped coatings are common (10-50%)
	Coarse Fragments:	not evident,
	Segregations:	not evident,
	Roots:	many (25-100/10x10cm) (Root size <1 mm),
	Soil fauna:	Activity is nil
	Cracks/Macropores:	Cracks are evident(width 10-20 mm), macropores are nil
	Moisture/Consistence:	dry,
	Erodibility Tests:	Crumb (EAT) test showed aggregates slake,
	Field chemical tests:	Field pH is 8.5 (Raupach), HCl test showed no effervescence, H2O2 showed effervescence
	Sample taken:	disturbed
	Layer Notes:	45 degrees shear planes evident.
**Layer 3**	Horizon: B22k (carbonates)	
1.40 - 2.30 m	Texture:	heavy clay
	Colour:	dark reddish brown (5YR 3/3) [moist] with 0% unspecified mottles
	Structure:	strong pedality (prismatic, 20 - 50 mm, fabric is smooth-faced peds), ped coatings are common (10-50%)
	Coarse Fragments:	sedimentary, many (20-50%), sedimentary, rounded, gravel (6-20 mm),
	Segregations:	common (10% - 20%), calcareous,
	Roots:	common (10-25/10x10cm) (Root size <1 mm),
	Soil fauna:	Activity is nil
	Cracks/Macropores:	Cracks are evident(width 10-20 mm), macropores are nil
	Moisture/Consistence:	moderately moist,
	Erodibility Tests:	Crumb (EAT) test showed aggregates slake,
	Field chemical tests:	Field pH is 10.0 (Raupach), HCl test showed no effervescence,
	Sample taken:	disturbed
	Layer Notes:	45 degrees shear planes evident. Gravel type = basalt.
**Layer 4**	Horizon: Dk (carbonates)	
2.30 - 7.00 m	Texture:	coarse clayey sand
	Colour:	strong brown (bright brown) (7.5YR 5/6) [moist] with 0% unspecified mottles
	Structure:	massive (fabric is earthy), ped coatings are none

	Coarse Fragments:	sedimentary, very abundant (> 90%), sedimentary, rounded, gravel (6-20 mm),
	Segregations:	many (20% - 50%), calcareous,
	Roots:	common (10-25/10x10cm) (Root size <1 mm),
	Soil fauna:	Activity is nil
	Cracks/Macropores:	Cracks are none(width 10-20 mm), macropores are nil
	Moisture/Consistence:	dry,
	Erodibility Tests:	Crumb (EAT) test showed aggregates slake,
	Field chemical tests:	Field pH is 10.0 (Raupach), HCl test showed no effervescence, H_2O_2 showed effervescence
	Sample taken:	disturbed
	Layer Notes:	Varied sand and gravel layers. Gravel type = basalt.
**Layer 5**	Horizon: 2Dk (carbonates)	
7.00 - 9.00 m	Texture:	medium clay
	Colour:	strong brown (brown) (7.5YR 4/6) [moist] with 2% - 10% prominent unspecified grey mottles
	Structure:	strong pedality (fabric is smooth-faced peds), ped coatings are common (10-50%)
	Coarse Fragments:	common (10-20%), sedimentary, rounded, gravel (6-20 mm),
	Segregations:	abundant (> 50%), calcareous,
	Roots:	common (10-25/10x10cm) (Root size <1 mm),
	Soil fauna:	Activity is nil
	Cracks/Macropores:	Cracks are none(width 10-20 mm), macropores are nil
	Moisture/Consistence:	moist,
	Erodibility Tests:	Crumb (EAT) test showed aggregates slake,
	Field chemical tests:	Field pH is 10.0 (Raupach), HCl test showed no effervescence,
	Sample taken:	disturbed
	Layer Notes:	45 degrees shear planed evident Gravel type = basalt. Overlies discountinuous travertine layer

LABORATORY TESTS

None available

F

Report generated on 11/12/2019 at 09:18 PM

Table S2: Site level treatment according to season

Location	Altitude (m)	Landuse	Land Manageme nt Regime	Practice	Tillage	Fallow	Season	Crop Type
Willow Tree	330	Grazing	NP	Native	None	None	Winter '99	Grasseses
Willow Tree	330	Grazing	NP	Native	None	None	Winter '99	Grasseses
Blackville	360	Grazing	NP	Native	None	None	Winter '99	Grasseses
Blackville	360	Grazing	NP	Native	None	None	Winter '99	Grasses/clovers
Bonny Ridge	340	Grazing	IP	Introduced	None	None	Winter '99	Grasses
Bonny Ridge	340	Grazing	IP	Introduced	None	None	Winter '99	Lucerne
Blackville	461	Grazing	IP	Introduced	None	None	Winter '99	Lucerne
Blackville	364	Grazing	IP	Introduced	None	None	Winter '99	Grasses
Willow Tree	360	Cropping	SFZ	Rotation	Zero	Short	Winter '99	Wheat
Willow Tree	360	Cropping	SFZ	Rotation	Zero	Short	Winter '99	Wheat
Quirindi	330	Cropping	SFZ	Rotation	Zero	Short	Winter '99	Fallow
Quirindi	330	Cropping	SFZ	Rotation	Zero	Short	Winter '99	Fallow
Willow Tree	340	Cropping	SFC	Rotation	Conventional	Short	Winter '99	Wheat
Willow Tree	340	Cropping	SFC	Rotation	Conventional	Short	Winter '99	Wheat
Willow Tree	340	Cropping	SFC	Rotation	Conventional	Short	Winter '99	Fallow
Willow Tree	340	Cropping	SFC	Rotation	Conventional	Short	Winter '99	Fallow
Blackville	380	Cropping	SFC	Rotation	Conventional	Short	Winter '99	Wheat
Blackville	380	Cropping	SFC	Rotation	Conventional	Short	Winter '99	Fallow
Spring Ridge	330	Cropping	LFZ	Rotation	Zero	Long	Winter '99	Faba beans
Spring Ridge	330	Cropping	LFZ	Rotation	Zero	Long	Winter '99	Fallow
Quirindi	330	Cropping	LFC	Rotation	Conventional	Long	Winter '99	Wheat
Quirindi	330	Cropping	LFC	Rotation	Conventional	Long	Winter '99	Fallow
Blackville	360	Cropping	LFC	Rotation	Conventional	Long	Winter '99	Fallow
Blackville	360	Cropping	LFC	Rotation	Conventional	Long	Winter '99	Wheat
Willow Tree	330	Grazing	NP	Native	None	None	Spring '99	Grasseses
Willow Tree	330	Grazing	NP	Native	None	None	Spring '99	Grasseses
Blackville	360	Grazing	NP	Native	None	None	Spring '99	Grasseses
Blackville	360	Grazing	NP	Native	None	None	Spring '99	Grasses/clovers
Bonny Ridge	340	Grazing	IP	Introduced	None	None	Spring '99	Grasses
Bonny Ridge	340	Grazing	IP	Introduced	None	None	Spring '99	Lucerne
Blackville	461	Grazing	IP	Introduced	None	None	Spring '99	Lucerne
Blackville	364	Grazing	IP	Introduced	None	None	Spring '99	Grasses
Willow Tree	360	Cropping	SFZ	Rotation	Zero	Short	Spring '99	Wheat
Willow Tree	360	Cropping	SFZ	Rotation	Zero	Short	Spring '99	Wheat
Quirindi	330	Cropping	SFZ	Rotation	Zero	Short	Spring '99	Fallow
Quirindi	330	Cropping	SFZ	Rotation	Zero	Short	Spring '99	Fallow
Willow Tree	340	Cropping	SFC	Rotation	Conventional	Short	Spring '99	Wheat
Willow Tree	340	Cropping	SFC	Rotation	Conventional	Short	Spring '99	Wheat
Willow Tree	340	Cropping	SFC	Rotation	Conventional	Short	Spring '99	Fallow
Willow Tree	340	Cropping	SFC	Rotation	Conventional	Short	Spring '99	Fallow
Blackville	380	Cropping	SFC	Rotation	Conventional	Short	Spring '99	Wheat
Blackville	380	Cropping	SFC	Rotation	Conventional	Short	Spring '99	Fallow
Spring Ridge	330	Cropping	LFZ	Rotation	Zero	Long	Spring '99	Faba beans
Spring Ridge	330	Cropping	LFZ	Rotation	Zero	Long	Spring '99	Corn
Quirindi	330	Cropping	LFC	Rotation	Conventional	Long	Spring '99	Wheat
Quirindi	330	Cropping	LFC	Rotation	Conventional	Long	Spring '99	Fallow
Blackville	360	Cropping	LFC	Rotation	Conventional	Long	Spring '99	Fallow
Blackville	360	Cropping	LFC	Rotation	Conventional	Long	Spring '99	Wheat
Willow Tree	330	Grazing	NP	Native	None	None	Summer '99	Grasseses
Willow Tree	330	Grazing	NP	Native	None	None	Summer '99	Grasseses
Blackville	360	Grazing	NP	Native	None	None	Summer '99	Grasseses
Blackville	360	Grazing	NP	Native	None	None	Summer '99	Grasses/clovers
Bonny Ridge	340	Grazing	IP	Introduced	None	None	Summer '99	Grasses
Bonny Ridge	340	Grazing	IP	Introduced	None	None	Summer '99	Lucerne
Blackville	461	Grazing	IP	Introduced	None	None	Summer '99	Lucerne
Blackville	364	Grazing	IP	Introduced	None	None	Summer '99	Grasses
Willow Tree	360	Cropping	SFZ	Rotation	Zero	Short	Summer '99	Fallow
Willow Tree	360	Cropping	SFZ	Rotation	Zero	Short	Summer '99	Fallow
Quirindi	330	Cropping	SFZ	Rotation	Zero	Short	Summer '99	Sorghum
Quirindi	330	Cropping	SFZ	Rotation	Zero	Short	Summer '99	Sorghum
Willow Tree	340	Cropping	SFC	Rotation	Conventional	Short	Summer '99	Fallow
Willow Tree	340	Cropping	SFC	Rotation	Conventional	Short	Summer '99	Fallow
Willow Tree	340	Cropping	SFC	Rotation	Conventional	Short	Summer '99	Sunflower
Willow Tree	340	Cropping	SFC	Rotation	Conventional	Short	Summer '99	Sunflower
Blackville	380	Cropping	SFC	Rotation	Conventional	Short	Summer '99	Fallow
Blackville	380	Cropping	SFC	Rotation	Conventional	Short	Summer '99	Sorghum
Spring Ridge	330	Cropping	LFZ	Rotation	Zero	Long	Summer '99	Fallow

Spring Ridge	330	Cropping	LFZ	Rotation	Zero	Long	Summer '99	Corn
Quirindi	330	Cropping	LFC	Rotation	Conventional	Long	Summer '99	Fallow
Quirindi	330	Cropping	LFC	Rotation	Conventional	Long	Summer '99	Sorghum
Blackville	360	Cropping	LFC	Rotation	Conventional	Long	Summer '99	Sorghum
Blackville	360	Cropping	LFC	Rotation	Conventional	Long	Summer '99	Fallow
Willow Tree	330	Grazing	NP	Native	None	None	Autumn '00	Grasseses
Willow Tree	330	Grazing	NP	Native	None	None	Autumn '00	Grasseses
Blackville	360	Grazing	NP	Native	None	None	Autumn '00	Grasseses
Blackville	360	Grazing	NP	Native	None	None	Autumn '00	Grasses/clovers
Bonny Ridge	340	Grazing	IP	Introduced	None	None	Autumn '00	Grasses
Bonny Ridge	340	Grazing	IP	Introduced	None	None	Autumn '00	Lucerne
Blackville	461	Grazing	IP	Introduced	None	None	Autumn '00	Lucerne
Blackville	364	Grazing	IP	Introduced	None	None	Autumn '00	Grasses
Willow Tree	360	Cropping	SFZ	Rotation	Zero	Short	Autumn '00	Fallow
Willow Tree	360	Cropping	SFZ	Rotation	Zero	Short	Autumn '00	Fallow
Quirindi	330	Cropping	SFZ	Rotation	Zero	Short	Autumn '00	Sorghum
Quirindi	330	Cropping	SFZ	Rotation	Zero	Short	Autumn '00	Sorghum
Willow Tree	340	Cropping	SFC	Rotation	Conventional	Short	Autumn '00	Fallow
Willow Tree	340	Cropping	SFC	Rotation	Conventional	Short	Autumn '00	Fallow
Willow Tree	340	Cropping	SFC	Rotation	Conventional	Short	Autumn '00	Sunflower
Willow Tree	340	Cropping	SFC	Rotation	Conventional	Short	Autumn '00	Sunflower
Blackville	380	Cropping	SFC	Rotation	Conventional	Short	Autumn '00	Faba beans
Blackville	380	Cropping	SFC	Rotation	Conventional	Short	Autumn '00	Sorghum
Spring Ridge	330	Cropping	LFZ	Rotation	Zero	Long	Autumn '00	Fallow
Spring Ridge	330	Cropping	LFZ	Rotation	Zero	Long	Autumn '00	Corn
Quirindi	330	Cropping	LFC	Rotation	Conventional	Long	Autumn '00	Fallow
Quirindi	330	Cropping	LFC	Rotation	Conventional	Long	Autumn '00	Sorghum
Blackville	360	Cropping	LFC	Rotation	Conventional	Long	Autumn '00	Sorghum
Blackville	360	Cropping	LFC	Rotation	Conventional	Long	Autumn '00	Fallow
Willow Tree	330	Grazing	NP	Native	None	None	Winter '00	Grasseses
Willow Tree	330	Grazing	NP	Native	None	None	Winter '00	Grasseses
Blackville	360	Grazing	NP	Native	None	None	Winter '00	Grasseses
Blackville	360	Grazing	NP	Native	None	None	Winter '00	Grasses/clovers
Bonny Ridge	340	Grazing	IP	Introduced	None	None	Winter '00	Grasses
Bonny Ridge	340	Grazing	IP	Introduced	None	None	Winter '00	Lucerne
Blackville	461	Grazing	IP	Introduced	None	None	Winter '00	Lucerne
Blackville	364	Grazing	IP	Introduced	None	None	Winter '00	Grasses
Willow Tree	360	Cropping	SFZ	Rotation	Zero	Short	Winter '00	Fallow
Willow Tree	360	Cropping	SFZ	Rotation	Zero	Short	Winter '00	Fallow
Quirindi	330	Cropping	SFZ	Rotation	Zero	Short	Winter '00	Fallow
Quirindi	330	Cropping	SFZ	Rotation	Zero	Short	Winter '00	Fallow
Willow Tree	340	Cropping	SFC	Rotation	Conventional	Short	Winter '00	Fallow
Willow Tree	340	Cropping	SFC	Rotation	Conventional	Short	Winter '00	Fallow
Willow Tree	340	Cropping	SFC	Rotation	Conventional	Short	Winter '00	Wheat
Willow Tree	340	Cropping	SFC	Rotation	Conventional	Short	Winter '00	Wheat
Blackville	380	Cropping	SFC	Rotation	Conventional	Long	Winter '00	Barley
Blackville	380	Cropping	SFC	Rotation	Conventional	Long	Winter '00	Fallow
Spring Ridge	330	Cropping	LFZ	Rotation	Zero	Long	Winter '00	Wheat
Spring Ridge	330	Cropping	LFZ	Rotation	Zero	Long	Winter '00	Faba beans
Quirindi	330	Cropping	LFC	Rotation	Conventional	Long	Winter '00	Fallow
Quirindi	330	Cropping	LFC	Rotation	Conventional	Long	Winter '00	Fallow
Blackville	360	Cropping	LFC	Rotation	Conventional	Long	Winter '00	Fallow
Blackville	360	Cropping	LFC	Rotation	Conventional	Long	Winter '00	Fallow
Willow Tree	330	Grazing	NP	Native	None	None	Spring '00	Grasseses
Willow Tree	330	Grazing	NP	Native	None	None	Spring '00	Grasseses
Blackville	360	Grazing	NP	Native	None	None	Spring '00	Grasseses
Blackville	360	Grazing	NP	Native	None	None	Spring '00	Grasses/clovers
Bonny Ridge	340	Grazing	IP	Introduced	None	None	Spring '00	Grasses
Bonny Ridge	340	Grazing	IP	Introduced	None	None	Spring '00	Lucerne
Blackville	461	Grazing	IP	Introduced	None	None	Spring '00	Lucerne
Blackville	364	Grazing	IP	Introduced	None	None	Spring '00	Grasses
Willow Tree	360	Cropping	SFZ	Rotation	Zero	Short	Spring '00	Fallow
Willow Tree	360	Cropping	SFZ	Rotation	Zero	Short	Spring '00	Fallow
Quirindi	330	Cropping	SFZ	Rotation	Zero	Short	Spring '00	Fallow
Quirindi	330	Cropping	SFZ	Rotation	Zero	Short	Spring '00	Fallow
Willow Tree	340	Cropping	SFC	Rotation	Conventional	Short	Spring '00	Fallow
Willow Tree	340	Cropping	SFC	Rotation	Conventional	Short	Spring '00	Fallow
Willow Tree	340	Cropping	SFC	Rotation	Conventional	Short	Spring '00	Wheat
Willow Tree	340	Cropping	SFC	Rotation	Conventional	Short	Spring '00	Wheat
Blackville	380	Cropping	SFC	Rotation	Conventional	Long	Spring '00	Barley
Blackville	380	Cropping	SFC	Rotation	Conventional	Long	Spring '00	Fallow

Spring Ridge	330	Cropping	LFZ	Rotation	Zero	Long	Spring '00	Wheat
Spring Ridge	330	Cropping	LFZ	Rotation	Zero	Long	Spring '00	Faba beans
Quirindi	330	Cropping	LFC	Rotation	Conventional	Long	Spring '00	Fallow
Quirindi	330	Cropping	LFC	Rotation	Conventional	Long	Spring '00	Fallow
Blackville	360	Cropping	LFC	Rotation	Conventional	Long	Spring '00	Fallow
Blackville	360	Cropping	LFC	Rotation	Conventional	Long	Spring '00	Fallow
Willow Tree	330	Grazing	NP	Native	None	None	Summer'00	Grasseses
Willow Tree	330	Grazing	NP	Native	None	None	Summer'00	Grasseses
Blackville	360	Grazing	NP	Native	None	None	Summer'00	Grasseses
Blackville	360	Grazing	NP	Native	None	None	Summer'00	Grasses/clovers
Bonny Ridge	340	Grazing	IP	Introduced	None	None	Summer'00	Grasses
Bonny Ridge	340	Grazing	IP	Introduced	None	None	Summer'00	Lucerne
Blackville	461	Grazing	IP	Introduced	None	None	Summer'00	Lucerne
Blackville	364	Grazing	IP	Introduced	None	None	Summer'00	Grasses
Willow Tree	360	Cropping	SFZ	Rotation	Zero	Short	Summer'00	Sorghum
Willow Tree	360	Cropping	SFZ	Rotation	Zero	Short	Summer'00	Sorghum
Quirindi	330	Cropping	SFZ	Rotation	Zero	Short	Summer'00	Sorghum
Quirindi	330	Cropping	SFZ	Rotation	Zero	Short	Summer'00	Sorghum
Willow Tree	340	Cropping	SFC	Rotation	Conventional	Short	Summer'00	Sorghum
Willow Tree	340	Cropping	SFC	Rotation	Conventional	Short	Summer'00	Sorghum
Willow Tree	340	Cropping	SFC	Rotation	Conventional	Short	Summer'00	Sorghum
Willow Tree	340	Cropping	SFC	Rotation	Conventional	Short	Summer'00	Sorghum
Blackville	380	Cropping	SFC	Rotation	Conventional	Short	Summer'00	Fallow
Blackville	380	Cropping	SFC	Rotation	Conventional	Short	Summer'00	Sorghum
Spring Ridge	330	Cropping	LFZ	Rotation	Zero	Long	Summer'00	Fallow
Spring Ridge	330	Cropping	LFZ	Rotation	Zero	Long	Summer'00	Fallow
Quirindi	330	Cropping	LFC	Rotation	Conventional	Long	Summer'00	Sorghum
Quirindi	330	Cropping	LFC	Rotation	Conventional	Long	Summer'00	Fallow
Blackville	360	Cropping	LFC	Rotation	Conventional	Long	Summer'00	Fallow
Blackville	360	Cropping	LFC	Rotation	Conventional	Long	Summer'00	Fallow
Willow Tree	330	Grazing	NP	Native	None	None	Autumn '01	Grasseses
Willow Tree	330	Grazing	NP	Native	None	None	Autumn '01	Grasseses
Blackville	360	Grazing	NP	Native	None	None	Autumn '01	Grasseses
Blackville	360	Grazing	NP	Native	None	None	Autumn '01	Grasses/clovers
Bonny Ridge	340	Grazing	IP	Introduced	None	None	Autumn '01	Grasses
Bonny Ridge	340	Grazing	IP	Introduced	None	None	Autumn '01	Lucerne
Blackville	461	Grazing	IP	Introduced	None	None	Autumn '01	Lucerne
Blackville	364	Grazing	IP	Introduced	None	None	Autumn '01	Grasses
Willow Tree	360	Cropping	SFZ	Rotation	Zero	Short	Autumn '01	Sorghum
Willow Tree	360	Cropping	SFZ	Rotation	Zero	Short	Autumn '01	Sorghum
Quirindi	330	Cropping	SFZ	Rotation	Zero	Short	Autumn '01	Sorghum
Quirindi	330	Cropping	SFZ	Rotation	Zero	Short	Autumn '01	Sorghum
Willow Tree	340	Cropping	SFC	Rotation	Conventional	Short	Autumn '01	Sorghum
Willow Tree	340	Cropping	SFC	Rotation	Conventional	Short	Autumn '01	Sorghum
Willow Tree	340	Cropping	SFC	Rotation	Conventional	Short	Autumn '01	Sorghum
Willow Tree	340	Cropping	SFC	Rotation	Conventional	Short	Autumn '01	Sorghum
Blackville	380	Cropping	SFC	Rotation	Conventional	Short	Autumn '01	Fallow
Blackville	380	Cropping	SFC	Rotation	Conventional	Short	Autumn '01	Sorghum
Spring Ridge	330	Cropping	LFZ	Rotation	Zero	Long	Autumn '01	Fallow
Spring Ridge	330	Cropping	LFZ	Rotation	Zero	Long	Autumn '01	Fallow
Quirindi	330	Cropping	LFC	Rotation	Conventional	Long	Autumn '01	Sorghum
Quirindi	330	Cropping	LFC	Rotation	Conventional	Long	Autumn '01	Fallow
Blackville	360	Cropping	LFC	Rotation	Conventional	Long	Autumn '01	Fallow
Blackville	360	Cropping	LFC	Rotation	Conventional	Long	Autumn '01	Fallow

Table S3: Mean abundance (standard error of mean) of invertebrate taxa recorded in pitfall traps (total number per field), across land use and agricultural management regimes. Each data point was the total abundance of 20 pitfall traps per field. Each field was sampled eight times (once each season over two years), the number of replicates for each land use type or management regime is dependent on groupings). For each taxa, where there were significant differences (P < 0.05). Within rows and category, means followed by different letters were significantly different at P < 0.05. Comparison of mean (Tukey's HSD) were completed within the category of land use type, and agricultural management regimes. NP = native pasture, IP = introduced pasture, LFZ = long fallow zero tillage, SFZ = short fallow zero tillage, LFC = long fallow conventional tillage, SFC = short fallow conventional tillage.

**Taxa**	**Land Use Type**		**Management Regimes**					
	**Grazing**	**Cropping**	**NP**	**IP**	**LFZ**	**SFZ**	**LFC**	**SFC**
Formicidae	383.2a	157.0b	517.8a	248.6ab	206.6ab	181.9b	109.7b	155.3b
(Ants)	(70.6)	(16.7)	(133)	(36.3)	(72.3)	(35)	(36.3)	(24.4)
Iridomyrmex	191.0a	57.5b	359.3a	22.8b	24.5b	59.2b	96.5b	41.3b
	(54.4)	(8.1)	(101)	(5.6)	(15.7)	(17.4)	(22.1)	(8.1)
Pheidole	118.6a	32.5b	128a	109ab	120ab	22.2bc	5.4c	28.2bc
	(22.3)	(9.4)	(38.8)	(22.5)	(71.4)	(4.9)	(2.9)	(3.8)
Diptera	72.3	88.0	82.2ab	62.4b	111.9ab	47.8b	121.7a	84.5ab
	(10.0)	(7.1)	(15.0)	(8.2)	(20.5)	(5.7)	(8.2)	(13.1)
Rhytidoponera.	64.1a	63.0a	20.8bc	107.5a	59.8ab	97.3ab	0.84c	82.6ab
	(13.5)	(11.3)	(5.3)	(24.3)	(16.5)	(28.7)	(0.3)	(21.0)
Coleoptera	45.1b	100.7a	53.7b	36.5b	74.5ab	67.7ab	106.7a	127.5a
	(5.5)	(17.1)	(9.8)	(4.8)	(13.7)	(18.3)	(18.5)	(42.0)
Orthoptera	32.8a	20.7b	39.0a	26.7b	23.5b	23.5b	18.1b	19.7b
	(4.4)	(3.1)	(6.8)	(5.5)	(9.6)	(5.6)	(7.3)	(4.7)
Araneae	29.0a	20.0b	23.8ab	34.2a	20.5b	16.4b	23.7ab	19.9b
	(3.1)	(1.7)	(3.4)	(5.0)	(4.4)	(1.9)	(4.4)	(2.8)
Hemiptera	24.7	14.8	20.3	29.1	9.1	4.8	36.6	8.8
	(8.2)	(7.7)	(8.2)	(14.2)	(3.4)	(1.1)	(30.8)	(1.7)
Acarina	18.2a	7.9b	8.7b	27.8a	2.3b	5.3b	8.3b	11.4b
	(4.3)	(3.2)	(1.7)	(8.1)	(0.9)	(1.8)	(2.8)	(8.2)
Hymenoptera	9.5a	7.4a	9.7ab	9.3ab	10.4ab	3.8b	13.3a	5.0b
(excluding ants)	(1.0)	(1.2)	(1.4)	(1.4)	(4.1)	(0.8)	(3.8)	(0.7)
Diplopoda	4.4	2.9	3.0	5.8	2.7	6.2	1.7	1.6
	(2.3)	(0.7)	(1.7)	(4.4)	(0.8)	(2.4)	(0.7)	(0.3)
Isopoda	3.5a	1.2b	1.6ab	5.5a	0.6b	1.2b	1.5ab	1.3b
	(1.2)	(0.2)	(0.7)	(2.3)	(0.5)	(0.5)	(0.6)	(0.3)
Dermaptera	2.3b	5.2a	0.2b	4.3ab	1.6ab	6.3ab	6.4a	6.3a
	(0.7)	(0.9)	(0.1)	(1.4)	(0.6)	(0.9)	(1.8)	(1.9)
Thysanoptera	2.2	2.0	3.5	0.8	4.1	1.9	2.4	1.1
	(0.7)	(0.4)	(1.4)	(0.3)	(2.5)	(0.6)	(0.8)	(0.3)
Blattodea	0.6 b	3.9a	1.2bc	0.1c	9.3a	6.2ab	1.1bc	2.5abc
	(0.2)	(0.9)	(0.4)	(0.04)	(4.7)	(2.5)	(0.3)	(0.5)
Chilopoda	0.5	1.1	0.2	0.8	1.1	1.3	0.4	1.3
	(0.1)	(0.2)	(0.1)	(0.2)	(0.4)	(0.4)	(0.1)	(0.4)
Mecoptera	0.1	0.2	0.03	0.09	1.1	0.0	0.2	0.2
	(0.3)	(1.6)	(0.03)	(0.07)	(1.1)	(0.0)	(0.2)	(0.1)

Table S4: Mean abundance (standard error of mean) of invertebrate taxa recorded in pitfall traps (total number per field), across season. Each data point was the total abundance of 20 pitfall traps per field. Each field was sampled once in each of the four seasons and repeated in the following calendar year, n = 48). Within rows and category, means followed by different letters were significantly different at P < 0.05. Comparison of mean (Tukey's HSD) were completed within the category of season. Also, within rows, means followed by different letters were significantly different at P < 0.05.

	**Season**			
**Taxa**	**Autumn**	**Winter**	**Spring**	**Summer**
Formicidae	192.9b	100.3b	180.5b	455.8a
(Ants)	(23.2)	(21.3)	(24.3)	(93.8)
Diptera	104.6ab	131.6a	51.3bc	44.9c
	(14.6)	(13.9)	(13.9)	(13.9)
Rhytidoponera	76.2a	26.6b	72.9a	77.8a
	(19.4)	(7.6)	(19.2)	(19.9)
Iridomyrmex	68.2b	30.6b	61.3b	248a
	(14.8)	(9.0)	(12.7)	(70.9)
Pheidole	59.3b	42.8b	45.2b	120a
	(8.6)	(16.8)	(8.1)	(33.8)
Orthoptera	53.6a	2.2c	17.bc	26.3b
	(6.8)	(0.4)	(2.4)	(5.0)
Coleoptera	39.2b	134.8a	93.6ab	61.1ab
	(5.0)	(42.6)	(13.3)	(9.9)
Hemiptera	25.1	8.3	30.0	9.0
	(10.7)	(2.1)	(20.5)	(2.4)
Araneae	21.8a	13.3b	27.7a	29.4a
	(4.2)	(4.0)	(4.0)	(4.0)
Dermaptera	10.3a	0.8b	3.1b	2.8b
	(2.1)	(0.2)	(0.9)	(0.5)
Hymenoptera (excluding ants)	9.1ab	3.0c	13.6a	6.7bc
	(0.9)	(0.7)	(2.8)	(1.0)
Acarina	4.7b	24.4a	5.5b	10.9ab
	(0.9)	(9.7)	(1.2)	(2.3)
Diplopoda	4.2	6.2	2.3	0.88
	(1.4)	(3.2)	(0.6)	(0.2)
Blattodea	3.8	1.6	4.1	1.9
	(1.3)	(0.6)	(1.9)	(0.4)
Thysanoptera	1.3	2.4	3.2	1.3
	(0.7)	(0.7)	(1.0)	(0.4)
Chilopoda	0.9ab	0.5b	1.6a	0.5b
	(0.3)	(0.3)	(0.3)	(0.1)
Isopoda	0.5	1.6	2.6	3.2
	(0.2)	(0.5)	(0.8)	(1.4)
Mecoptera	0.0	0.2	0.5	0.04
	(0.0)	(0.1)	(0.4)	(0.03)
